# How to rescue misfolded SERT, DAT and NET: targeting conformational intermediates with atypical inhibitors and partial releasers

**DOI:** 10.1042/BST20180512

**Published:** 2019-05-07

**Authors:** Shreyas Bhat, Amy Hauck Newman, Michael Freissmuth

**Affiliations:** 1 Institute of Pharmacology and the Gaston H. Glock Research Laboratories for Exploratory Drug Development, Center of Physiology and Pharmacology, Medical University of Vienna, Vienna, Waehringerstrasse 13a, Vienna, Austria; 2 Molecular Targets and Medications Discovery Branch, National Institute on Drug Abuse, Intramural Research Program, Baltimore, MD 21224, U.S.A.

**Keywords:** dopamine transporter, pharmacochaperoning, protein folding, serotonin transporter

## Abstract

Point mutations in the coding sequence for solute carrier 6 (SLC6) family members result in clinically relevant disorders, which are often accounted for by a loss-of-function phenotype. In many instances, the mutated transporter is not delivered to the cell surface because it is retained in the endoplasmic reticulum (ER). The underlying defect is improper folding of the transporter and is the case for many of the known dopamine transporter mutants. The monoamine transporters, i.e. the transporters for norepinephrine (NET/SLC6A2), dopamine (DAT/SLC6A3) and serotonin (SERT/SLC6A4), have a rich pharmacology; hence, their folding-deficient mutants lend themselves to explore the concept of pharmacological chaperoning. Pharmacochaperones are small molecules, which bind to folding intermediates with exquisite specificity and scaffold them to a folded state, which is exported from the ER and delivered to the cell surface. Pharmacochaperoning of mutant monoamine transporters, however, is not straightforward: ionic conditions within the ER are not conducive to binding of most typical monoamine transporter ligands. A collection of compounds exists, which are classified as atypical ligands because they trap monoamine transporters in unique conformational states. The atypical binding mode of some DAT inhibitors has been linked to their anti-addictive action. Here, we propose that atypical ligands and also compounds recently classified as partial releasers can serve as pharmacochaperones.

## Introduction

Transporters for the monoamines norepinephrine (NET), serotonin (SERT) and dopamine (DAT) belong to the SLC6 (solute carrier 6) transporter family. All SLC6 transporters are Na^+^ and substrate symporters: the electrochemical gradient of Na^+^ powers the uphill uptake of monoamines and allows for their intracellular accumulation [1]. Expression of these monoamine transporters in the adult central nervous system (CNS) is limited to cognate monoaminergic neurons, where they are delivered to both the somatodendritic and the axonal compartments [2–5]. The physiological role of somatodendritic monoamine transporters is poorly understood [5]. In the axons, monoamine transporters are predominantly concentrated at the rim of the synapse [2–4]; this perisynaptic localisation is thought to limit the overflow of released neurotransmitters [6]. Monoamine transporters are crucial for refilling of the presynaptic vesicles. This is achieved by a relay, where the sequential action of plasmalemmal transporters and of vesicular monoamine transporters (VMATs) retrieves monoamines and shields them from degradation by monoamine oxidase and catechol-O-methyltransferase. Presynaptic monoamine transporters, therefore, shape synaptic transmission by both, modulating the signal [6] and maintaining vesicular stores. The importance of monoamine transporters for vesicular refilling can be appreciated by examining the phenotype from loss-of-function mutations in the human DAT. The affected children suffer from a syndrome of dystonia and Parkinsonism [7–9]. These symptoms reflect dopamine deficiency rather than exaggerated dopaminergic stimulation. In fact, genetic ablation of DAT in mice also severely compromises vesicular dopamine stores: they are reduced by ∼95% [10].

**Figure 1.**
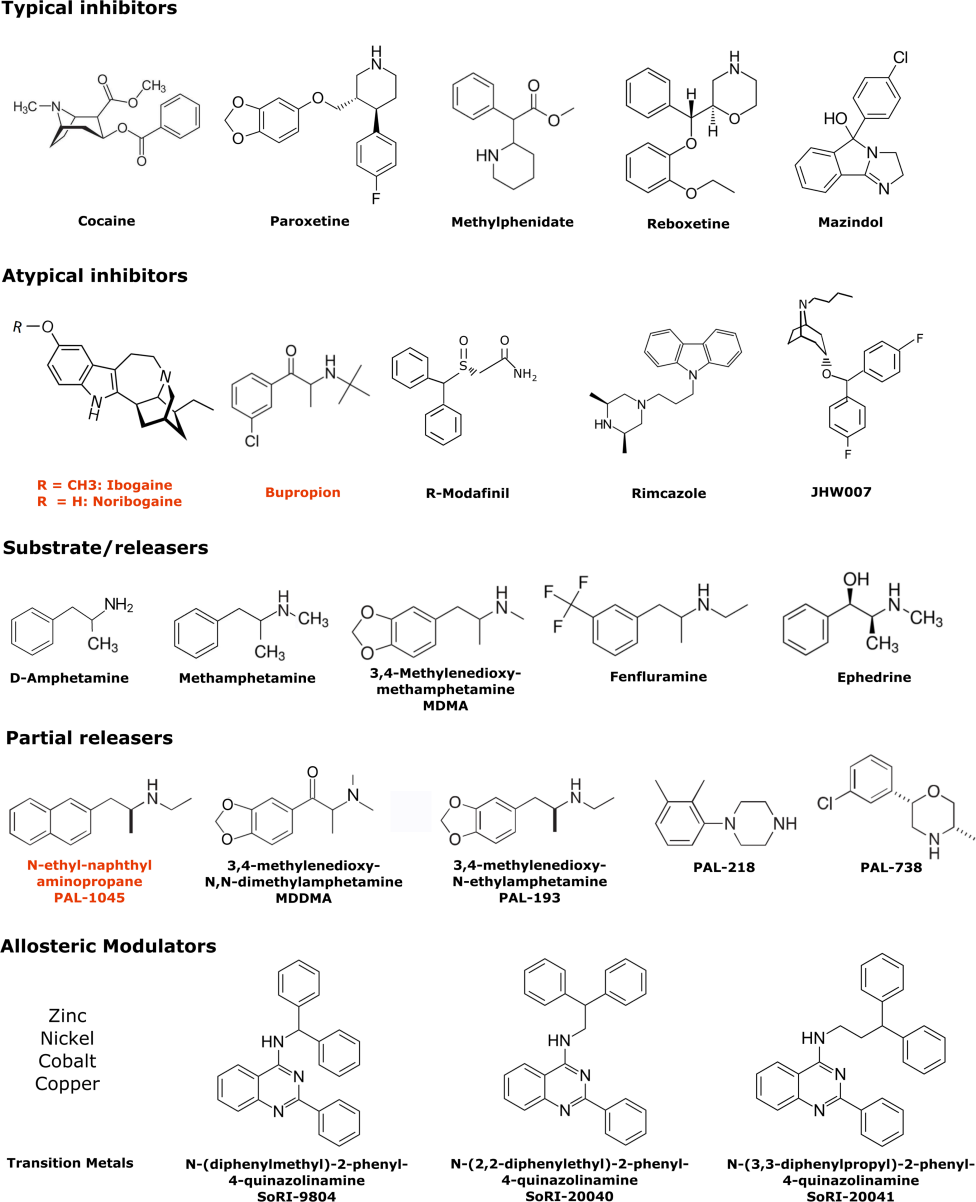
Extending the pharmacology of monoamine transporters beyond the currently existing dichotomy. Exogenous ligands that bind to monoamine transporters have been divided into non-transportable typical reuptake inhibitors and transportable substrates/releasers. Typical inhibitors exert their psychostimulant and addictive properties by binding to monoamine transporters in an outward-open state thereby blocking transporter function. However, a few atypical inhibitors for monoamine transporters have been discovered that have reduced addictive and locomotor properties when compared with cocaine. This ligand class is thought to stabilise more of an occluded or inward-open transporter states. Substrates that induce neurotransmitter efflux through monoamine transporters are termed as releasers. Some substrates have reduced efficacy in inducing such neurotransmitter release and are termed as ‘partial releasers’. There also exist ligands that selectively modulate either uptake, release or both through monoamine transporters in an allosteric manner. Compound names highlighted in red have additionally been shown to act as pharmacochaperones, i.e. they improve trafficking and rescue function of ER-retained transporters arising from point mutations.

Fröhlich and Loewi discovered that cocaine potentiated of the action of adrenaline already at the beginning of the 20th century [11], but it took another 50 years until it was appreciated that transporter-mediated reuptake rather than enzymatic degradation was the predominant mechanism for terminating neurotransmission [12–14]. Indeed, cocaine and tricyclic antidepressants were instrumental as tools to define the reuptake process [12,14]. For obvious economic reasons, the chemical space of monoamine transporter ligands has been explored by both the pharmaceutical industry and the illicit market (Figure 1). Hence, the pharmacology of monoamine transporters is extremely rich and presumably unrivalled by any other drug target. Inhibitors of SERT and NET are effective in the treatment of major depression; selective serotonin reuptake inhibitors (SSRIs) are also useful in the management of generalised anxiety disorder and obsessive–compulsive disorder. DAT inhibitors, such as methylphenidate and the DAT substrate, amphetamine, are clinically used in the treatment of attention deficit hyperactivity disorders (ADHD) and modafinil and its R-enantiomer (armodafinil) are clinically approved for management of sleep disorders but also prescribed off-label for treatment of ADHD [15]. Inhibition of dopamine reuptake at DAT, by drugs of abuse such as cocaine, is highly rewarding, rapidly raising extracellular dopamine levels in the nucleus accumbens and leading some to dependence. This can also be achieved by amphetamine and its derivatives (including the synthetic cathinones), which trigger reverse transport by DAT, SERT and NET with variable specificity [16]. Accordingly, ligands are classified as inhibitors, which typically bind to the outward-facing conformation of the monoamine transporters, or as releasers, which are substrates capable of triggering the exchange mode [16]. However, several classes of atypical DAT inhibitors have been described over the past 25 years that inhibit the reuptake of dopamine but do not engender cocaine-like reinforcing properties and indeed, can block the behavioural effects of cocaine and methamphetamine [17,18]. Furthermore, it has been appreciated that another class of compounds differ from typical releasers in their efficacy. Because they induce less release than amphetamine and other full releasers, they are referred to as partial releasers [19]. Partial releasers are attractive because they occupy the transporter rendering the binding site unavailable for amphetamine or any other full releaser. Because a partial releaser produces less dopamine efflux than amphetamine [20], its rewarding effects are limited and thus it is another potential candidate for treatment of psychostimulant abuse disorders [18]. Chemical structures of representative members of these classes of drugs are shown in Figure 1. We argue here that such atypical inhibitors and partial releasers are also of interest because of their binding mode: it allows for rescuing misfolded transporters.

**Figure 2.**
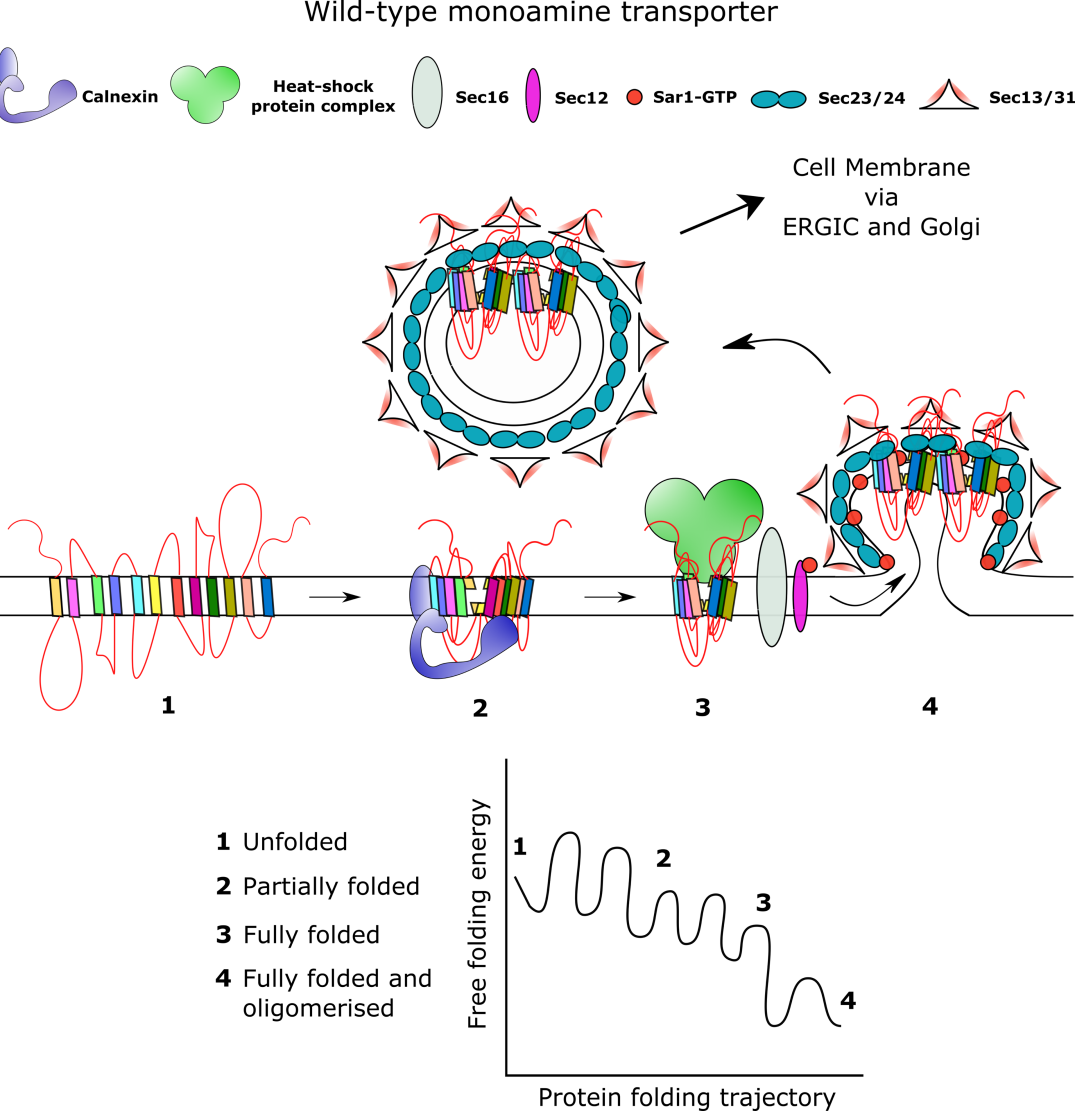
Native folding and trafficking of monoamine transporters from ER to the plasma membrane. On insertion of the complete monoamine transporter protein onto the ER membrane, the transporter undergoes folding under the influence of conditions imposed by the cytosol, ER membrane and ER lumen. Initially present in a high-energy unfolded state (denoted as **1**), the transporter undergoes N-linked glycosylation by the oligosaccharyl transferase (OST) enzyme complexes (not shown in the figure). Transporters tagged with N-linked glycans are recognised by calnexin (denoted as **2**), an important folding sensor that resides in the ER membrane. Calnexin and other chaperone proteins allow for efficient folding and quaternary protein complex formation by preventing offset folding pathways that lead to protein aggregation. Release of folding intermediates from calnexin leads to trimming of the terminal glucose by α-glucosidase II and expose protein sites that facilitates oligomerization. The cytosolic part of the protein, in the meantime, engages with the HSPs relay (denoted as **3**). Release of HSPs exposes the Sec23/24-binding site (denoted as **4**) and allows the transporter to be recruited into the nascent coat protein complex II (COPII)-coated vesicle. These containers initiate anterograde trafficking of transporter oligomers to the plasma membrane through the ERGIC and the Golgi apparatus.

**Figure 3.**
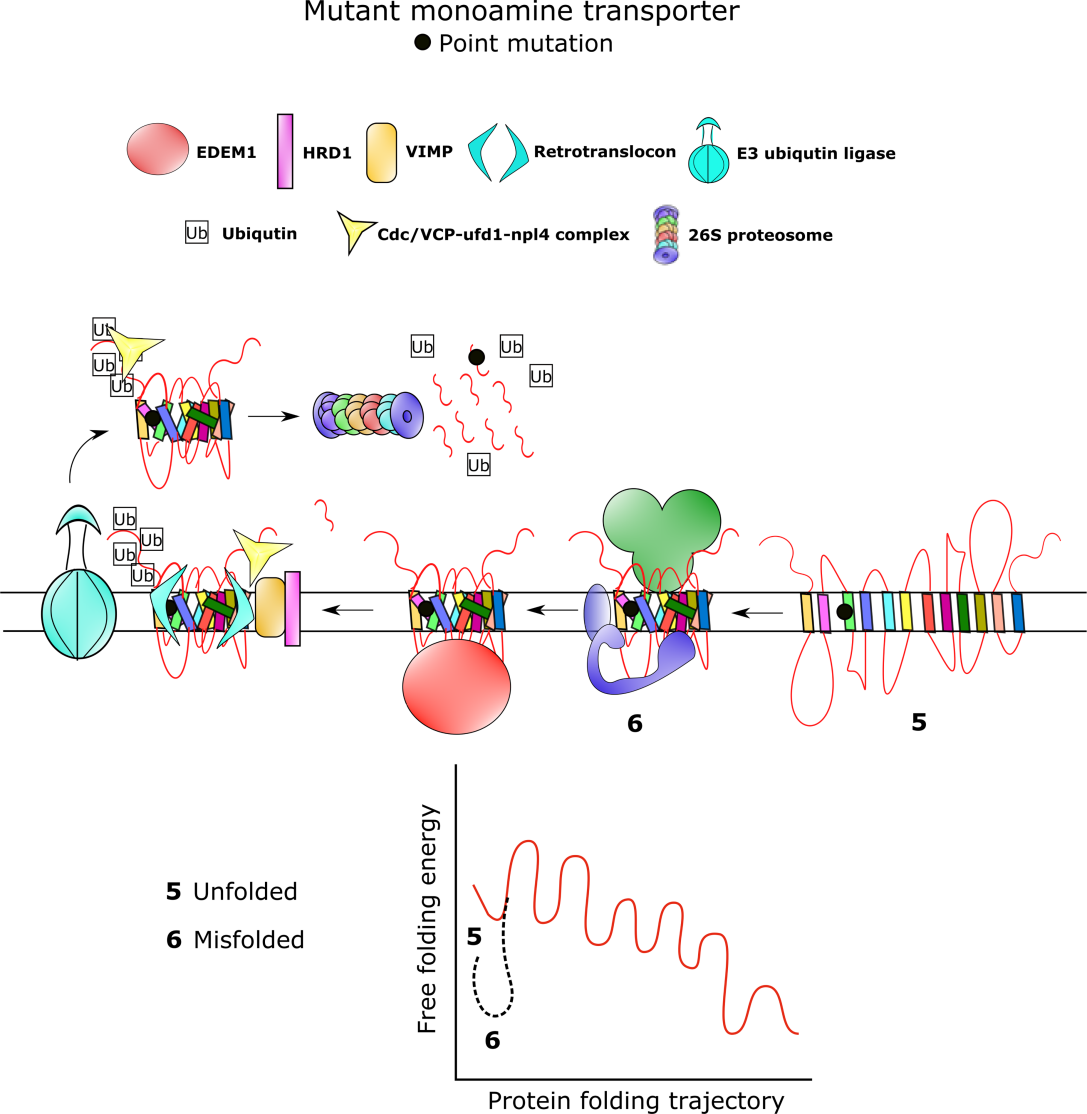
Most monoamine transporter mutations lead to impaired trafficking of the transporters to the plasma membrane. Point mutations in monoamine transporters often lead to ER retention of the transporters. In most instances, this is due to protein misfolding. The stringent quality control exerted by the ER prevents trafficking of non-natively folded proteins (denoted as **5**) to the plasma membrane. Transporter mutants reach a premature local minimum energy state (denoted as **6**) due to improper folding. This prevents them to progress along the folding trajectory. Consequently, glycosylation of the stalled mutant protein by the folding sensor UDP-glucose:glycoprotein glucosyltransferase (UGGT) effectively reverses the action of α-glucosidase II. This UGGT action not only prevents anterograde trafficking of the transporters via the COPII machinery but also increases the likelihood of re-association of the transporter mutants with calnexin. Calnexin-bound misfolded transporters are subsequently targeted to the ERAD pathway either through a E3 ubiquitin ligase or by the ER degradation-enhancing α-mannosidase-like protein (EDEM1). These proteins facilitate entry of misfolded transporters from the ER membrane to the cytosol through the retrotranslocon pathway. Ubiquitin-binding proteins such as the Cdc48/VCP-Npl4-Ufd1 complex direct transporters to the 26S proteasome where they undergo proteasomal degradation.

Like most membrane proteins, SLC6 transporters are assembled in the ER before they are delivered to the plasma membrane (Figure 2). There are at least 60 different mutations in various SLC6 human transporter genes, which result in misfolding of the protein and subsequent ER retention (Figure 3). Loss of transporter function often leads to clinically relevant disorders [21,22]. Fourteen of these mutations occur in the human DAT; they give rise to infantile/juvenile dystonia and Parkinsonism [6–8]. A folding-deficient mutation in the NET (NET-A457P) results in postural hypotension [23,24]. The rich pharmacology of monoamine transporters provides a treasure trove in the search for pharmacochaperones, i.e. small molecules, which bind to and stabilise folding intermediates and thus allow progress in an otherwise stalled folding trajectory. Thus, we suggest that insights obtained in rescuing mutated monoamine transporters can also guide the development of strategies, which can rescue other folding-deficient SLC6 transporters.

## A rational approach to chaperoning with small molecules

**Figure 4.**
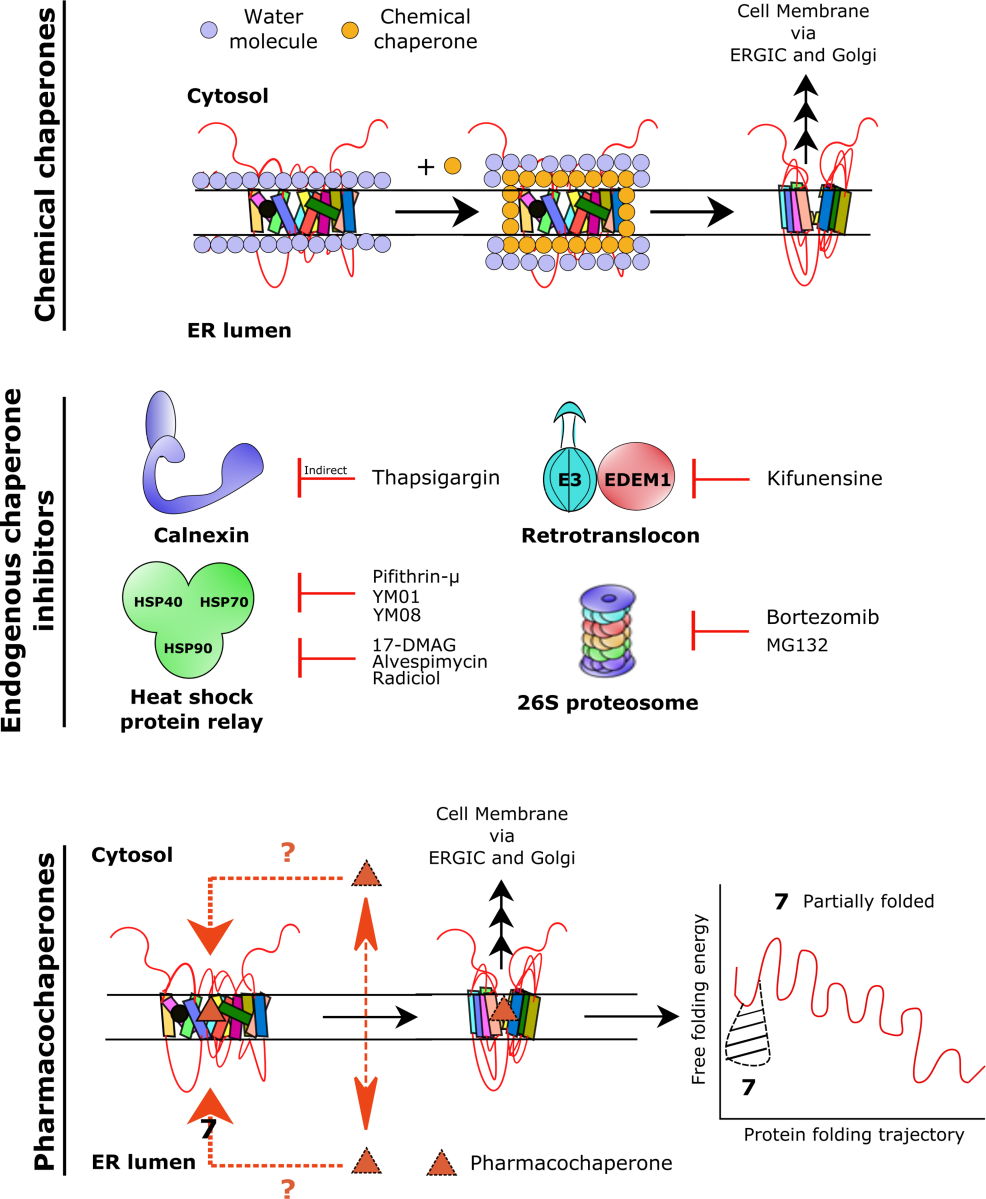
Mechanisms for correction of misfolded proteins associated with monoamine transporter mutations. Chemical chaperones (**top** panel) facilitate protein folding by e.g. mitigating the effect of free water. Prominent examples include osmolytes (e.g. betaine), glycerol and DMSO. The mechanism of action of sodium phenylbutyrate (4-PBA) is poorly defined. Inhibitors of endogenous chaperones and of the degradation machinery (**middle** panel) relax ER quality control. This can lead to improved ER export and plasma membrane trafficking of mutant transporters. Pharmacochaperones (**bottom** panel) specifically bind to mutant transporters (either at an orthosteric or allosteric binding site). Pharmacochaperones reduce energy barrier imposed by mutations (denoted as **7**) and facilitate folding of protein mutants from a misfolded state to a partially or completely folded state. This leads to rescue of transporter function. Pharmacochaperones for monoamine transporter mutants include the atypical inhibitors ibogaine, noribogaine, bupropion and the partial releaser PAL-1045 (see Figure 1).

Many drugs have been discovered, which remedy defects in protein folding, including those arising from mutations in membrane proteins [25]. At least three approaches can be envisaged to enhance the folding of membrane proteins by small molecules within the endoplasmic reticulum (ER). This allows for the subsequent entry of the corrected protein into the secretory pathway, i.e. anterograde trafficking via the ER–Golgi intermediate compartment (ERGIC) and the Golgi apparatus to reach their target compartment (the plasma membrane, the lysosome, etc.): (i) addition of osmolytes or organic molecules (collectively referred to as chemical chaperones) such as DMSO, glycerol and betaine can facilitate folding in a non-specific manner (Figure 4, top); (ii) agents, which inhibit the activity of endogenous proteinaceous chaperones, can relax quality control in the ER and favour anterograde trafficking of the mutant protein over its elimination by ER-associated degradation (ERAD) (Figure 4, middle); (iii) ligands, which bind to the protein of interest, can also act as pharmacological chaperones or pharmacochaperones, if they interact with folding intermediates in the conformational search space (Figure 4, bottom). This interaction smoothens the energy landscape of the folding trajectory in a manner analogous to proteinaceous chaperones and facilitates the progression to a folded state suitable for ER export. Pharmacochaperones confer certain advantages over other small molecules [26]. One is exquisite selectivity. Pharmacochaperones selectively target the mutant protein of interest as opposed to inhibitors of the folding machinery; the latter is likely to result in untoward, off-target effects [27].

There are two examples of pharmacochaperones, which have been approved for the treatment of affected patients. Migalastat, an inhibitor of α-galactosidase A (α-GalA), corrects the folding defect in several mutated versions of α-galactosidase A, which cause Fabry's disease [28]. Lumacaftor (and recently tezacaftor) corrects the folding deficit by the ΔF508 mutation in the cystic fibrosis transmembrane conductance regulator (CFTR/ABCC7) gene [29]. CFTR-ΔF508 is the most frequent mutation occurring in patients suffering from cystic fibrosis. These compounds have been identified by screening approaches. We propose that a conceptual framework is useful to guide the search for pharmacochaperones and other small molecules, which restore the function of misfolded monoamine transporters and other SLC6 transporters. The chaperone/COPII (coat protein complex II) exchange model takes into account the sequential interactions, which the transporter undergoes until it is exported from the ER [30]. This model is mainly based on studying the biogenesis of SERT and posits the following sequence of events (Figure 2). (i) After exiting the SEC61 translocon, the nascent transporter is subject to core glycosylation, which supports the interaction with calnexin. Calnexin does not only bind via the sugar moieties by its lectin domain; its transmembrane helix for shielding the transmembrane domain of the transporter [31]. In this stage, SERT is monomeric [32]. (ii) The folding state of SERT is also monitored by a cytosolic heat shock protein (HSP) relay [33]. Accordingly, inhibitors of HSP90 and of HSP70 increase surface expression of folding-deficient SERT mutants [33]. This is also true for folding-deficient DAT mutants [34,35]. HSP70 binds to the proximal portion of the C-terminus of SERT; this binding site is adjacent to the site required for interaction with SEC24C, the cargo receptor of the COPII [36]. Hence, recruitment of SEC24C and subsequent incorporation of the SERT into nascent COPII-coated vesicles is contingent on prior release of the HSP-relay. The proximal segment of SERT contains an amphipathic helix, which forms a salt bridge with the first intracellular loop and thus supports the annular arrangement of the 12 transmembrane helices [37]. Binding of HSP70 to the C-terminus of SERT, therefore, allows for monitoring progress in the folding trajectory. Inhibitors of HSP restore ER export of some—but not all—folding-deficient mutants of SERT [33] and of DAT [34,35] presumably by relaxing stringent quality control in the ER. This allows for their escape from ERAD. Finally, it is worth noting that these experiments relied on heterologous expression of transporters. However, they can be—in part—recapitulated in cells, which endogenously express SERT: inhibition of HSP90 enhances cell surface levels of and substrate uptake by SERT in the human choriocarcinoma cell line JAR, which harbour the endogenous transporter [33].

The following observations support the assumption that this model is also relevant for SLC6 transporters other than monoamine transporters: (i) The C-terminal SEC24-binding site is also present in transporters from other branches, i.e. in the GABA-transporter 1/SLC6A1 [38], the glycine transporter 1/SLC6A9, the betaine/GABA-transporter 1/SLC6A12 and the GABA-transporter 4/SLC6A11 [39], the glycine transporter 2/SLC6A5 [40] and the amino acid transporter B^0,+^/SLC6A14 [41]. (ii) Mutations in the glycine transporter 2 (GlyT2), which give rise to startle disease/hyperekplexia, can be transmitted in both, a dominant and a recessive manner [40,42,43]. (iii) Misfolded GlyT2-S512R can be rescued by chemical chaperones and by 4-phenylbutyrate. 4-Phenylbutyrate is thought to exert its action by affecting the expression of HSP70 isoforms, which shifts the balance from ERAD to folding [44,45]. 4-Phenylbutyrate also rescues folding-deficient mutants of SERT [46] and of the creatine transporter 1/SLC6A8 [47]; surface expression of several disease-associated creatine transporter 1 mutants can also be restored by inhibition of HSP90 and HSP70 [47].

The HSP/COPII exchange model provides a framework to understand why some mutants are dominant negative and others are transmitted in a recessive manner, i.e. individuals are only affected if they are homozygous [30]. ER export of the GABA-transporter 1 [48,49], of DAT [50] and of SERT [51] is contingent on their assembly into oligomers. This is presumably true for most, if not all, SLC6 transporters. In fact, the mutant variant NET-A457P retains wild-type NET in the ER [24]. This dominant negative action accounts for its autosomal dominant transmission of the mutant allele. The recessive transmission of mutations can be rationalised by the fact that the folding-deficient mutants are stalled in a chaperone complex prior to oligomer formation. This conjecture is supported by the observation that, in the presence of the dominant mutant GlyT2-S512R, overexpression of calnexin allows for the escape of wild-type GlyT2 from the ER. Retention of the misfolded GlyT2-S512R by calnexin shields the folded wild-type transporter from oligomerization with GlyT2-S512R [40]. The HSP/COPII exchange model also explains why manipulations of the HSP-relay can—in some instances—rescue folding-deficient mutants and why inhibition of HSP's can synergize with pharmacochaperones [33–35]. However, the HSP/COPII exchange model does not shed any light on the folding trajectory *per se* and does not explain why some transporter ligands act as pharmacochaperones, while others are ineffective. Progress requires an understanding of the conformations selected by pharmacochaperones, as the folding trajectory of membrane proteins is poorly understood [25].

## Discovery of pharmacochaperones for folding-deficient monoamine transporters

Pharmacochaperoning of monoamine transporters was discovered by serendipity: a mutation was introduced in SERT to disrupt the interaction with SEC24C; this mutation also impaired folding of the mutant protein. Of the three classes of SERT ligands that were tested (i.e. substrates, typical inhibitors and ibogaine), only ibogaine was effective in restoring ER export/cell surface delivery and function (i.e. substrate uptake and high-affinity inhibitor binding) to the mutant SERT [52]. Moreover, noribogaine, a primary metabolite of ibogaine (Figure 1), is more effective than ibogaine presumably due to its higher affinity to WT-SERT [33,53]. Ibogaine and noribogaine bind from the extracellular side of SERT [54] but stabilise the transporter in an inward-facing conformation [55,56]. The rescue by ibogaine indicates that the inward-facing conformation was one of the conformations visited by SERT during its folding trajectory. This conjecture was confirmed by examining the effect of mutations, which trap SERT in the inward-facing state [57,58]. These mutations were also effective in remedying the folding deficit [37]. However, individual folding-deficient mutations differed in their susceptibility to these second-site suppressors. Individual folding-deficient mutations also differed in the extent to which they were trapped in complex with the cytosolic HSP-relay and to which noribogaine and HSP inhibitors acted synergistically to restore folding [33]. Taken together, these observations demonstrated that individual mutations were stalled at different points of the folding trajectory.

In contrast with ibogaine and noribogaine, typical inhibitors of monoamine transporters such as cocaine, mazindol, paroxetine and reboxetine (Figure 1) bind to the outward-facing conformation and require Na^+^ for high-affinity binding. The ER does not contain any appreciable concentration of Na^+^. It is, therefore, not surprising that this class of compounds has proven ineffective in pharmacochaperoning folding-deficient SERT. In fact, when tested on disease-relevant folding-deficient mutants of DAT [6–8], typical inhibitors of DAT (e.g. cocaine and methylphenidate; Figure 1) did not rescue any of these mutants [59]. It is not clear, which conformational state is stabilised by substrates in the absence of Na^+^, but it is unlikely to be the inward-facing conformation: substrates dissociate rapidly from the inward-facing state at low Na^+^ concentrations [60]. In fact, serotonin, *p*-chloroamphetamine and amphetamine fail to rescue folding-deficient mutants of SERT [52] and of DAT [59], respectively. However, partial substrates and atypical inhibitors may be different: ibogaine binds in the absence of Na^+^ and renders the inner vestibule accessible to cysteine-modifying reagents [61]. Similarly, the partial substrate PAL-1045 binds with appreciable affinity to the inward-facing state in the absence of Na^+^[60]. Ibogaine and noribogaine also bind to DAT with appreciable affinity [53]. Accordingly, noribogaine also corrects the folding defect of some DAT mutants heterologously expressed in cells [34,35,59]. The efficacy of noribogaine suffices to restore DAT function *in vivo* in *Drosophila melanogaster* [34,35]. Bupropion was also effective in rescuing some DAT mutants [57]. Bupropion is an inhibitor of DAT derived from the substrate cathinone. It is likely to have a binding mode, which differs from that of typical inhibitors (see below). In total, only 5 of the 14 reported disease-relevant mutants [7–9] were amenable to rescue by ibogaine, noribogaine and bupropion. This indicates that, while some mutants are stalled in similar local minima, others are trapped at different points in the folding trajectory of DAT. Accordingly, a search is justified, which results in the expansion of the pharmacochaperone library not only to satisfy an unfulfilled medical need but also to better understand the folding landscape of DAT.

## Capitalising on monoamine transporter pharmacology to identify pharmacochaperones

**Figure 5.**
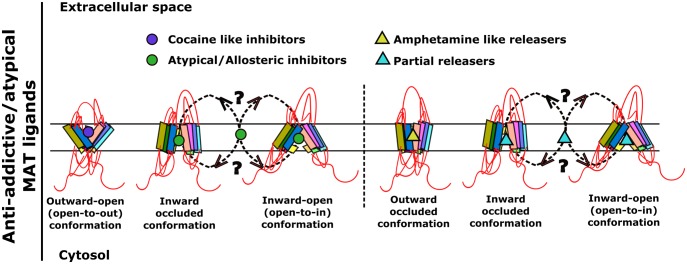
Proposed mechanism of action of atypical monoamine transporter ligands designed as medications to treat psychostimulant abuse and addiction. Atypical ligands targeting monoamine transporters are thought to stabilise transporter in unique conformational states, which differ from the outward-facing conformation stabilised by cocaine. For instance, SERT is trapped in the inward-open state by ibogaine and noribogaine. Similarly, bupropion and PAL-1045 bind preferentially to occluded and inward-open transporter states. Bupropion is the only drug that has been clinically approved for treating substance use disorders.

The rewarding and addictive properties of cocaine are caused by inhibition of DAT. However, it has been appreciated that inhibitors of DAT differ substantially in their reinforcing properties [62–64]. Compounds that have a delayed onset of action and hence, for kinetic reasons, [65] are less likely to produce an acute surge of dopamine in the nucleus accumbens are typically less reinforcing. In fact, some of these compounds antagonise the reinforcing actions of cocaine [64,66]. These compounds are classified as atypical DAT inhibitors [67]. Atypical DAT inhibitors are of potential interest in the treatment of psychostimulant use disorders. In fact, bupropion was the first drug shown to facilitate smoking cessation and nicotine abstinence in an adequately powered trial [68]. Examples of atypical reuptake inhibitors include congeners belonging to the 3α-diphenylmethoxytropane (especially JHW007) [66,69], rimcazole [70], ibogaine, bupropion, and modafinil classes of molecules (Figure 1). The pharmacokinetic differences do not necessarily explain their reduced abuse liability. Interestingly, atypical DAT inhibitors, at least in the benztropine and modafinil classes of compounds, appear to induce conformations of DAT, which differ from the outward-facing state (Figure 5, left) [71–74]. This is also evident for ibogaine and its congeners, which trap monoamine transporters in the inward-facing state [54–56]. However, the available evidence also suggests that atypical inhibitors differ in their binding mode. This conclusion is based on two approaches, i.e. by (i) comparing their affinity for DAT mutants, which are trapped in the outward and inward-facing state [71,75,76] and (ii) by determining the accessibility of cysteine residues to chemical modification [72]. It is worth noting that the list of atypical inhibitors includes the approved drugs modafinil and bupropion (Figure 1). Ibogaine, although not approved for clinical use, has been used for centuries by indigenous people in transition rites. As outlined above, bupropion, ibogaine and its metabolite noribogaine are effective pharmacochaperones for folding-deficient DAT variants, including the clinically relevant DAT mutants [34,35,59]. The safety profile of ibogaine congeners ought to be explored in a clinical setting to facilitate transition from recreational use into clinical trials.

It has also been appreciated that amphetamine-like releasers differ in their efficacy; the term partial releaser was coined to differentiate the compounds with reduced ability to trigger the substrate-exchange mode from those, which cause maximum efflux of monoamines [19,67]. Partial releasers trap the transporter in intermediate conformations (Figure 5, right), which must be transited during the transport cycle [19]. In fact, there appears to be a continuum between substrates, releasers and inhibitors: this conjecture is supported by the following observations: (i) cathinone is a releaser [16], but bupropion, a derivative of cathinone is an atypical inhibitor, (ii) 3,4-methylenedioxy-N,N-dimethylamphetamine (MDDMA) is a partial releaser, which can also adopt an inhibitory binding mode, its analogue 3,4-methylenedioxy-N,N,N-trimethylamphetamine (MDTMA) only binds in the inhibitory mode [77]. Partial releasers were generated by the systematic addition of functional groups to the backbone structures of phenethylamine, naphthyl-propane-amine and cathinone (Figure 1). Their original therapeutic application was directed toward the treatment of addiction [67]. Apart from their therapeutic potential, partial releasers are interesting tools. Because they trap monoamine transporters in distinct conformations, they can be used to interrogate the transport cycle and the folding trajectory. As a proof-of-principle, three naphthyl-propane-2-amine congeners were analysed for their action on SERT. This led to the identification of PAL-1045 ((S)-N-ethyl-1-(2-naphthyl)propane-2-amine), which is a partial releaser and dissociates slowly from the inward-facing state of SERT [60,78]. Thus, PAL-1045 binds SERT in the absence of Na^+^ [60]. As mentioned above, the ER lumen is devoid of appreciable Na^+^ concentrations; accordingly, the inward-facing state is the predicted minimum energy (=stable) conformation of monoamine transporters in the ER. Based on these interrelated properties (i.e. trapping of SERT in the inward-facing conformation and Na^+^-independent binding), PAL-1045 was predicted to be an effective pharmacochaperone. In fact, PAL-1045 rescued a folding-deficient mutant of SERT with an efficacy approaching that of noribogaine [78].

Pharmacochaperones that target the orthosteric binding site (i.e. the endogenous ligand binding site) have the disadvantage of also blocking the function of the rescued protein. In vivo, the limiting factor is the ratio of the half-lives of the rescued protein and of the pharmacochaperone: the refolded protein must persist after the pharmacochaperone has been eliminated. Migalastat, for instance, is administered every other day; this allows for unblocking the rescued α-galactosidase A [28]. Thus, the alternative is to assist folding by targeting allosteric binding sites. Binding of ligands to monoamine transporters is complex: (i) there is the substrate binding site, termed S1. In addition, there is a vestibular binding site, referred to as S2 [79]. There are conditions, where occupancy of the S1 and the S2 site is mutually non-exclusive [80]. (iii) The vestibule appears to contribute to a selectivity filter; ligands, which are selective for DAT or SET, differ in their on-rates rather than their off-rates [81]. (iv) DAT harbours a Zn^2+^-binding site on the extracellular side; individual transition metals exert distinct effects on DAT and discriminate between the forward and the reverse transport mode [82,83].

Taken together, these findings indicate that the vestibule of monoamine transporters can also accommodate small molecules, which act as allosteric modulators. In fact, allosteric modulators have been identified such as the quinazolinamines SoRI-9824, SoRI-20040 and SoRI-20041 (Figure 1). These can be either positive or negative allosteric modulators [84,85]. Their selective action on amphetamine-induced reverse transport can be rationalised by kinetic modelling; they are predicted to stabilise the inward-open apo- and/or substrate-bound states of monoamine transporters [19]. Thus, allosteric modulators are not only candidate compounds for the treatment of addiction [67] but they may also be effective pharmacochaperones.

## The challenge: structure–activity relationships for pharmacochaperoning

At the current stage, there are only a few starting points, which guide the rational search for pharmacochaperones. These include the chemical structures of effective pharmacochaperones (Figure 1, marked in red), and the inward-facing conformation of monoamine transporters, which is selected by both, (nor)ibogaine and PAL-1045 (see above). It is, however, clear that there must be additional conformational intermediates, which can be targeted. Progress is hampered by our poor understanding of the folding trajectory. Similarly, it is not clear, which conformations are selected/bound by atypical inhibitors such as bupropion, but it is evident that this must be one of many visited by the transporter during the folding trajectory of the protein within the ER. Hence, pharmacochaperones also provide a means to map the traps in the energy landscape of the folding trajectory: we posit that an orthogonal map of mutants versus effective pharmacochaperones will offer an insight into the conformational search space, which is traversed by the nascent transporter. Thus, pharmacochaperones are not only of interest because of their potential therapeutic application, but they also provide an approach to address the transporter folding problem.

**Perspectives**

- There are more than 60 disease-relevant mutations in SLC6 transporters, which result in folding deficiency. The phenotypic consequence of a folding defects, in most instances, a devastating disorder. Monoamine transporters have both a rich pharmacology and a large collection of folding-deficient mutants. Hence, they are well suited to explore the concept of pharmacochaperoning.
- At the current stage, there are only a few starting points, which guide the rational search for pharmacochaperones. These include the chemical structures of effective pharmacochaperones (Figure 1, marked in red), and the inward-facing conformation of monoamine transporters, which is selected by both, (nor)ibogaine and PAL-1045 (see above). It is, however, clear that there must be additional conformational intermediates, which can be targeted. Progress is hampered by our poor understanding of the folding trajectory. Similarly, it is not clear, which conformations are selected/bound by atypical inhibitors such as bupropion, but it is evident that this must be one of many visited by the transporter during the folding trajectory of the protein within the ER.
- A major future challenge is to extract the structure–activity relationship of pharmacochaperones. Pharmacochaperones provide a means to map the traps in the energy landscape of the folding trajectory: we posit that an orthogonal map of mutants versus effective pharmacochaperones will offer an insight into the conformational search space, which is traversed by the nascent transporter. Thus, pharmacochaperones are not only of interest because of their potential therapeutic application, but they also provide an approach to address the transporter folding problem.

## Abbreviations

CFTR: cystic fibrosis transmembrane conductance regulator
COPII: coat protein II
DAT: dopamine transporter
DMSO: dimethyl sulfoxide
ER: endoplasmic reticulum
ERAD: ER-associated protein degradation
ERGIC: ER–Golgi intermediate compartment
HSP: heat shock protein
Na^+^: sodium
NET: norepinephrine transporter
PAL-1045: (*S*)-*N*-ethyl-1-(2-naphthyl)propan-2-amine
SERT: serotonin transporter
SLC6: solute carrier 6
SRI-20040: *N*-(2,2-diphenylethyl)-2-phenyl-4-quinazolinamine
SRI-20041: *N*-(3,3-diphenylpropyl)-2-phenyl-4-quinazolinamine
SRI-9804: *N*-(diphenylmethyl)-2-phenyl-4-quinazolinamine
SSRIs: selective serotonin reuptake inhibitors
